# Comprehensive Pan-Cancer Analysis and the Regulatory Mechanism of ASF1B, a Gene Associated With Thyroid Cancer Prognosis in the Tumor Micro-Environment

**DOI:** 10.3389/fonc.2021.711756

**Published:** 2021-08-20

**Authors:** Jing Ma, Wei Han, Kai Lu

**Affiliations:** Department of Thyroid and Breast Surgery, Nanjing Hospital of Chinese Medicine affiliated to Nanjing University of Chinese Medicine, Nanjing, China

**Keywords:** anti-silencing function protein 1 homolog B (ASF1B), thyroid cancer, prognosis, pan-cancer, tumor immune micro-environment

## Abstract

**Background:**

The incidence of thyroid cancer, whose local recurrence and metastasis lead to death, has always been high and the pathogenesis of papillary thyroid carcinoma (PTC) has not been clearly elucidated. Therefore, the research for more accurate prognosis-related predictive biomarkers is imminent, and a key gene can often be a prognostic marker for multiple tumors.

**Methods:**

Gene expression profiles of various cancers in the TCGA and GTEx databases were downloaded, and genes significantly associated with the prognosis of THCA were identified by combining differential analysis with survival analysis. Then, a series of bioinformatics tools and methods were used to analyze the expression of the gene in each cancer and the correlation of each expression with prognosis, tumor immune microenvironment, immune neoantigens, immune checkpoints, DNA repair genes, and methyltransferases respectively. The possible biological mechanisms were also investigated by GSEA enrichment analysis.

**Results:**

656 differentially expressed genes were identified from two datasets and 960 DEGs that were associated with disease-free survival in THCA patients were screened *via* survival analysis. The former and the latter were crossed to obtain 7 key genes, and the gene with the highest risk factor, ASF1B, was selected for this study. Differential analysis of multiple databases showed that ASF1B was commonly and highly expressed in pan-cancer. Survival analysis showed that high ASF1B expression was significantly associated with poor patient prognosis in multiple cancers. In addition, ASF1B expression levels were found to be associated with tumor immune infiltration in THCA, KIRC, LGG, and LIHC, and with tumor microenvironment in BRCA, LUSC, STAD, UCEC, and KIRC. Further analysis of the relationship between ASF1B expression and immune checker gene expression suggested that ASF1B may regulate tumor immune patterns in most tumors by regulating the expression levels of specific immune checker genes. Finally, GSEA enrichment analysis showed that ASF1B high expression was mainly enriched in cell cycle, MTORC1 signaling system, E2F targets, and G2M checkpoints pathways.

**Conclusions:**

ASF1B may be an independent prognostic marker for predicting the prognosis of THCA patients. The pan-cancer analysis suggested that ASF1B may play an important role in the tumor micro-environment and tumor immunity and it has the potential of serving as a predictive biomarker for multiple cancers.

## Introduction

According to the American Cancer Society, 52,890 new cases of thyroid cancer were diagnosed in the United States in 2020, including 40,170 cases in women, which makes the thyroid cancer the fifth most common cancer among women in the United States (1). Papillary thyroid cancer (PTC) is a subtype of thyroid cancer and accounts for approximately 90% of all thyroid cancers (2). Although the incidence of PTC has been on the rise in recent years (2), PTC tends to be more differentiated and less malignant compared with other malignant tumors. Therefore, the majority of patients with highly differentiated PTC have a better overall prognosis with a 10-year survival rate greater than 90% (3, 4). However, a high rate of local recurrence is also a clinical feature of PTC, and studies have shown that high rates of lymph node metastasis and recurrence are important factors affecting the quality and duration of survival of PTC patients (5, 6). Therefore, further clarifying the molecular mechanisms of thyroid cancer and finding its key target molecules help the prediction of tumor prognosis.

Earlier studies have showed that despite its clinical heterogeneity, PTC has undergone consistent and specific molecular changes, and this finding may provide biomarkers for clinical applications (7). For example, Wreesmann et al. (8) found that abnormal MUC1 regulation was associated with aggressive behavior of PTC and may serve as a prognostic marker and potential therapeutic target for this disease. Min et al. (9) found that RARA, MAFF, miRNA-93, and their associated target gene SOX4, miRNA-342, and its target gene BCL2 were associated with the progression of TPC, and they may become prognostic markers and potential therapeutic targets for the disease. Xie et al (10) found that AHNAK2 could be used as a diagnostic and prognostic biomarker for PTC. With the advent of the Human Genome Project, high-throughput transcriptome data from cancer patients have become accessible because technologies have been developed to analyze large amounts of transcriptome data (11). Employing bioinformatics analysis with multiple databases to analyze gene expression, prognosis, mutation patterns, and function in different tumors is referred to as pan-cancer analysis. Thus, we can further use pan-cancer analysis to study the role of genes in different tumors and their association with different tumors.

In this study, we integrated THCA tumor samples from TCGA with normal samples from the GTEx database. Differential expression of mRNA was investigated using the Limma package of R software. One-way COX analysis was then used to screen genes associated with both THCA disease-free survival and DEGs: they were screened for overlapping genes in order to identify key genes. The expressions of these key genes were then analyzed in 33 cancers using pan-cancer analysis, and the correlation of the gene expressions with prognosis, immune microenvironment, immune checkpoint genes, and immune neo antigens were investigated.

## Material and Method

### Data Collection

All gene expression datasets and clinical information were obtained from the TCGA database (https://portal.gdc.cancer.gov/) and the GTEx database (https://gtexportal.org/) combined. mRNA expression profiles of 33 tumor cancer and normal tissue samples were downloaded from TCGA and GTEx, respectively. Removing batch effects from normalized data and corresponding to the corresponding clinical samples, removing duplicates and deleted samples from the downloaded data, and cases with missing clinical outcomes. There were 10201 tumor cases and 16571 controls in TCGA and GTEx datasets, details presented in **Supplementary Table 1**.Statistical analysis was performed using R software v4.0.3.

### Analysis of Differentially Expressed Genes (DEGs)

Differential expression of mRNA was studied using the Limma package of R software (version: 3.40.2). Adjusted P values were analyzed in TCGA or GTEx to correct for false-positive results. DEGs were obtained by screening with |log2(FC)| > 2, P< 0.05. The heat and volcano plots were plotted using the R package ggplot2.

### GO and KEGG Enrichment

To further explore the potential functions of potential targets, the data were analyzed by functional enrichment. Gene ontology (GO) is a widely used tool for annotating genes with functions, especially molecular functions (MF), biological pathways (BP), and cellular components (CC). To better understand the oncogenic role of target genes, the ClusterProfiler package in R was used to analyze the GO function of potential mRNAs and to enrich the KEGG pathway.

### Key Gene Screening

In addition to DEGs, the patient population was also screened for potential prognostic genes that affect DFS (disease-free survival) in THCA by dividing the patient population into two groups based on a median expression (high expression *vs*. low expression). After DEGs analysis and KM analysis, several significantly expressed genes in THCA were obtained and potential key genes were searched for through the “VennDiagram” in the R package.

### Survival Analysis

One-way COX was used to analyze the correlation between ASF1B expression and patient survival. The Kaplan-Meier(KM) method was used to compare the relationship between different ASF1B expression levels and the survival of patients. The expression levels of ASF1B in cancer tissues, paracancerous tissues, and non-cancerous tissues were divided into ASF1B high expression group and ASF1B low expression group. The One-way Cox survival analysis was performed using survival software, and the results were visualized by the “Forest plot” R package using forest plots.

### Immunological Correlation Analysis

Data on the scores of six immune infiltrating cells from 33 cancers were downloaded from the TIMER database, and the correlation between gene expression and these immune cell scores were analyzed separately. The immune scores and stromal scores of each individual tumor sample were analyzed using the R package ESTIMATE in order to observe gene expressions versus immune scores in 33 tumors.

### DNA Repair Genes and Methyltransferase Correlation Analysis

The correlation of five mis-match repair(MMRs) genes (MLH1, MSH2, MSH6, PMS2, EPCAM) with ASF1B expression was evaluated using expression profile data from TCGA. The correlation of the four methyltransferases’ expressions with ASF1B expression was analyzed. Visual analysis was performed using ggplot. The correlation was significant and positive when p < 0.05 and R> 0.20.

### Gene Set Enrichment Analysis (GSEA)

To observe the effect of gene expression on tumors, the samples were divided into two groups of high and low expression based on gene expression levels, and the enrichment of KEGG and HALLMARK pathways in the high and low expression group was analyzed using GSEA respectively.

## Results

### Results of Differentially Expressed Gene Screening in THCA

**Figure 1:**
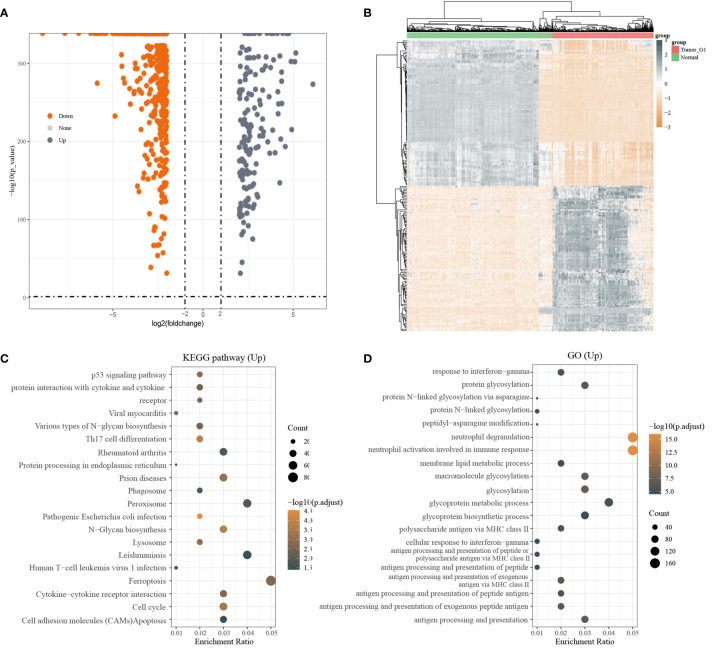
Differentially expressed genes and functional enrichment analysis; **(A)** volcano plot: grey dots indicate significantly differentially up-regulated genes and orange dots indicate significantly differentially down-regulated genes; **(B)** Heatmap plot of the DEGs; **(C)** KEGG enrichment analysis; **(D)** GO enrichment analysis

A total of 3331 up-regulated genes and 5695 down-regulated genes were obtained *via* the Limma package analysis of R software(|log2(FC)| > 0.3785, P< 0.05). Volcano plots were drawn using Fold change and corrected p-value values (**Supplementary Figure 1**). After screening with |log2(FC)| > 2, P< 0.05,160 up-regulated genes and 496 down-regulated genes were obtained (**Figure 1A**). The grey dots in the graph indicated significantly differentially up-regulated genes and the orange dots indicated significantly differentially down-regulated genes. Due to a large number of differential genes, only the top 50 up-regulated and down-regulated genes with the largest differential changes were shown here as heat maps, respectively (**Figure 1B**).

Next, KEGG pathway analysis was performed on differentially upregulated genes, which were involved in a total of 20 pathways that were mainly enriched for Ferroptosis(**Figure 1C**). GO enrichment analysis of differentially upregulated genes showed that they were mainly enriched in Neutrophil degranulation and Neutrophil activation that was involved in an immune response (**Figure 1D**).

### Screening of Prognosis-Related Genes in THCA

**Figure 2:**
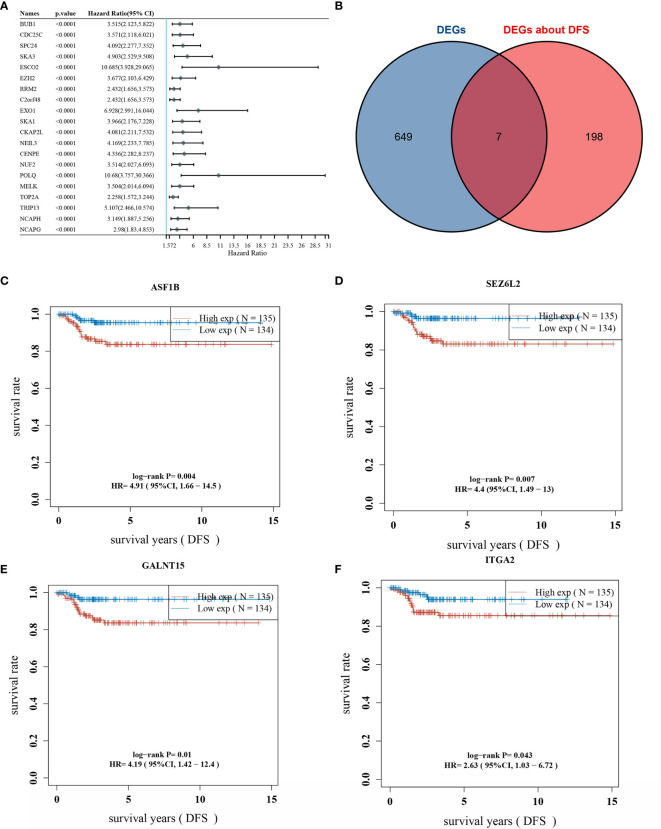
Overlap of differentially expressed genes and prognostic genes; **(A)** top 20 genes associated with DFS (disease-free survival) are shown; **(B)** overlap of DEGs and prognostic genes; **(C)** DFS survival curve of ASF1B; **(D)** DFS survival curve of SEZ6L2; **(E)** DFS survival curve of GALNT15; **(F)** DFS survival curve of ITGA2.

One-way COX analysis of genes in THCA yielded 960 genes associated with disease-free survival prognosis, The top 20 genes of statistically significance level were shown in **Figure 2A**. Seven key genes were obtained by crossing the 656 differentially expressed genes with the top 205 prognosis-associated genes (**Figure 2B**). Displaying the disease-free survival curves of these 7 genes yielded the gene with the highest risk factor: ASF1B, which was selected for subsequent correlation studies (**Figures 2C** **–F**). The gene lists of DEGs and Genes about DFS, their “Fold change” and P-values were included in **Supplementary Table 2**.

### Expression of ASF1B in THCA and Other Cancers

**Figure 3:**
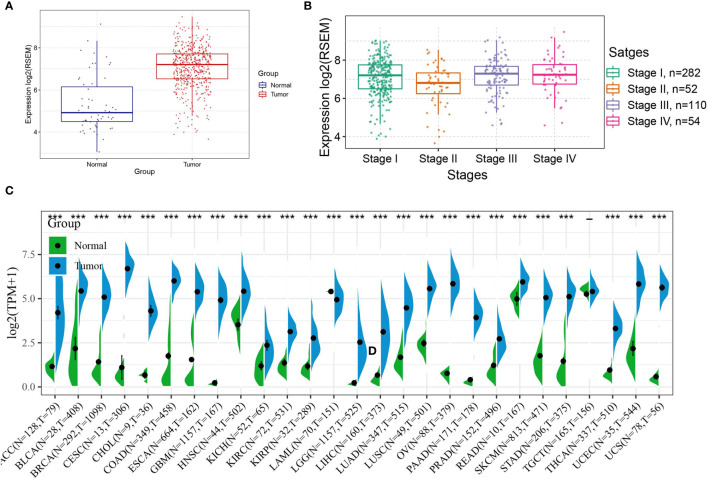
Expression of ASF1B in tumors; **(A)** Expression level of ASF1B in THCA; **(B)** Expression level of ASF1B in different TNM stages of THCA; **(C)** Expression level of ASF1B in 27 cancer type. In **(A)** *** is P < 0.0001.

First, the expression of ASF1B in THCA was observed, and the results showed that ASF1B expression levels were high in cancerous tissues (**Figure 3A**), the expression of ASF1B in different stage of THCA were shown in **Figure 3B**. Second, data from normal tissues in the GTEx database and data from TCGA tumor tissues were integrated to analyze the differences in ASF1B expression in 27 tumors. The results showed that ASF1B was highly expressed in all 26 tumors except TGCT, where the differences in ASF1B expression levels were not statistically significant compared with those in normal tissues (**Figure 3C**).

### Prognostic Analysis of ASF1B Expression in THCA and Other Cancers

**Figure 4:**
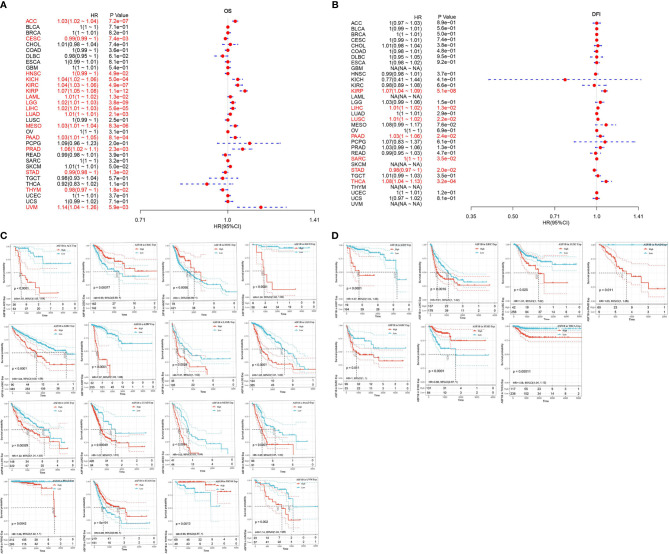
Univariate survival analysis was used to analyze the relationship between ASF1B expression and survival time in 33 tumors; **(A)** forest plot showing the relationship between ASF1B expression and OS; **(B)** forest plot showing the relationship between ASF1B expression and DFS; **(C)** KM curves of high and low ASF1B expression in 16 tumors significantly associated with OS survival; **(D)** KM curves of high and low ASF1B expression in 7 tumors significantly associated with DFS.

First, the association of ASF1B expression with overall survival and with disease-free survival in 33 TCGA tumors were calculated respectively by using univariate survival analysis. As shown in **Figure 4A**, ASF1B expression significantly affected the overall survival of ACC, CESC, HNSC, KICH, KIRC, KIRP, LAML, LGG, LIHC, LUAD, MESO, PAAD, PRAD, STAD, THYM, and UVM. The Kaplan-Meier curves were demonstrated in **Figure 4C**, and the results suggested that except for CESC, STAD, and THYM, high ASF1B expression was associated with poor patient prognosis. The association of ASF1B expression with disease-free survival was shown in **Figures 4B** **, D** and the results suggested that high ASF1B expression patient’ disease-free survival time was significantly lower than low ASF1B expression patient in KIRP, LIHC, LUSC, PAAD, SARC and THCA. Overall, the results suggested that ASF1B may be a potential prognostic predictor in THCA and other cancers.

### Correlation of ASF1B in THCA and Other Cancers With Tumor Immune Infiltration and Tumor Microenvironment

**Figure 5:**
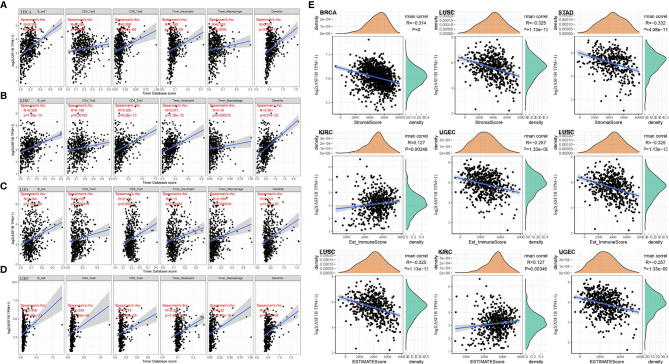
Correlation of ASF1B with tumor immune infiltration and the tumor microenvironment in THCA and other cancers; **(A)** Correlation of ASF1B expression with immune cell infiltration in THCA; **(B)** Correlation of ASF1B expression with immune cell infiltration in KIRC; **(C)** Correlation of ASF1B expression with immune cell infiltration in LGG; **(D)** Correlation of ASF1B expression with immune cell infiltration in LIHC; **(E)** Correlation of ASF1B with the immune score, stromal score, and ESTIMATE score in pan-cancer.

Whether ASF1B expression was correlated with the level of immune infiltration in THCA, or other different types of cancers has been investigated. Immune infiltration analysis showed that ASF1B expression was correlated with the level of immune infiltration in different types of tumors. In particular, in THCA, KIRC, LGG, and LIHC, ASF1B expression was significantly and positively correlated with the level of infiltration of B cells, CD4+ T cells, CD8+ T cells, Neutrophils, Macrophage, and Dendritic cells (**Figures 5A** **–D**). Via the R package for immune scoring and stromal scoring of individual tumor sample, as shown in **Figure 5E**, the top three tumors where ASF1B expression was significantly correlated with immune scoring were BRCA (R=-0.314, P<0.001), LUSC (R=-0.325, P<0.001), and STAD (R=-0.332, P<0.001); the top three tumors with stromal scoring (R=0.127, P<0.005), UCEC (R=-0.257, P<0.001), and LUSC (R=-0.325, P<0.001); the top three tumors significantly associated with stromal score were KIRC (R=0.127, P<0.005), UCEC (R=- 0.257, P<0.001) and LUSC (R=-0.325, P<0.001); the top three tumors significantly correlated with the composite score were LUSC, KIRC, and UCEC. Based on above results, it’s suggested that in terms of the tumor immune microenvironment, ASF1B expression levels were significantly negatively correlated with the immune score in BRCA, LUSC, STAD, UCEC while significantly positively correlated with the immune score in KIRC.

### Relationship Between ASF1B Expression and Immune Checkpoints and Immune Neoantigens

**Figure 6:**
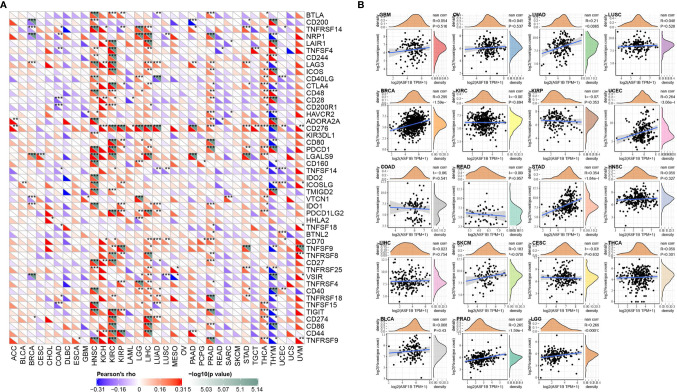
Correlation analysis of ASF1B expression in pan-cancer with immune neoantigens and immune checkpoint genes; **(A)** Correlation analysis of ASF1B expression in pan-cancer with immune checkpoint gene expression; **(B)** Correlation analysis of ASF1B expression in 19 tumors with the number of tumor neoantigens. In **(A)** * is P < 0.05, ** is P < 0.01 and *** is P < 0.001.

More than 40 common immune checkpoint genes were collected and the relationship between ASF1B expression and immune checkpoint gene expression was analyzed. The results were shown in **Figure 6A**. In a variety of tumors such as HNSC, KIRC, and LIHC, ASF1B expression was positively correlated with the expression levels of several immune checkpoint genes. It’s suggested that in these tumors, ASF1B may regulate the tumor immune pattern by regulating the expression levels of specific immune checkpoint genes. **Figure 6B** demonstrated the relationship between ASF1B expression and the number of neoantigens. The results found that ASF1B expression was significantly positively correlated with the number of neoantigens in LUAD, BRCA, UCEC, STAD, PRAD, and LGG.

### Relationship Between ASF1B Expression and DNA Repair Genes and Methyltransferase Expression

**Figure 7:**
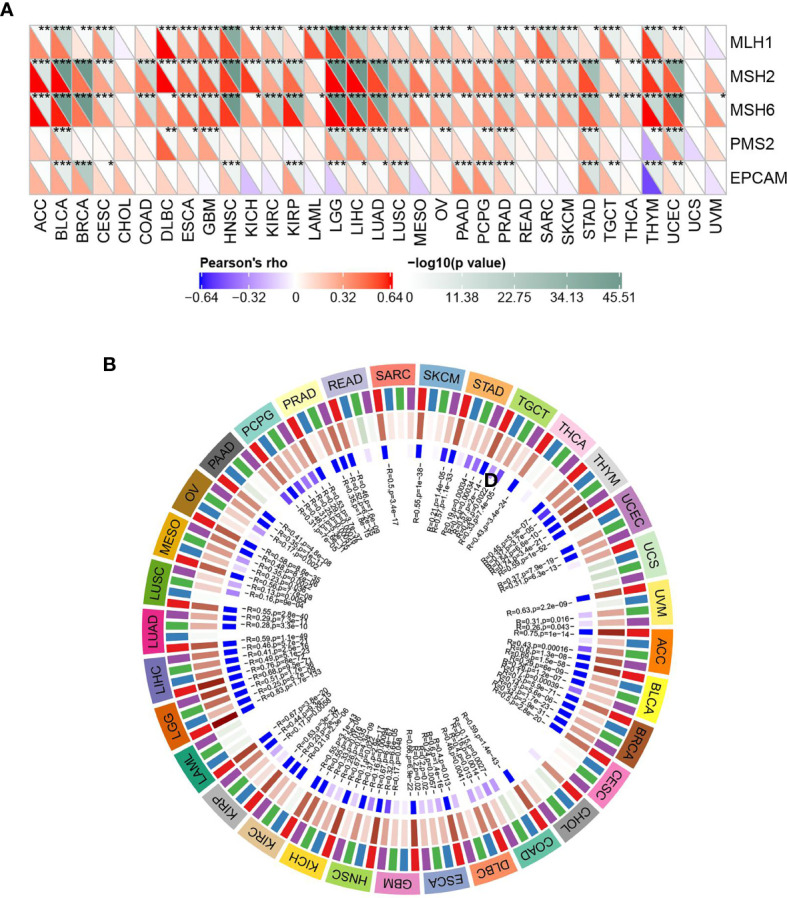
ASF1B expression in relation to DNA repair genes and methyltransferases; **(A)** correlation between ASF1B expression and gene expression levels of five MMRs; **(B)** correlation between ASF1B expression and expression of four methyltransferases; red: DNMT1, blue: DNMT2, green: DNMT3A, purple: DNMT3B. In **(A)** * is P < 0.05,** is P < 0.01, *** is P < 0.001.

As shown in **Figure 7A**, ASF1B expression was significantly positively correlated with DNA repair genes in all cancers except CHOL, LAML, UCS, and UVM. DNA methylation can cause changes in stain structure, DNA conformation, DNA stability, and DNA-protein interactions and thus control gene expression. As shown in **Figure 7B**, it’s found that ASF1B expression was significantly and positively correlated with methyltransferase in all cancer species except CHOL and UCS. It is suggested that ASF1B may indirectly influence cancer development and progression by regulating epigenetic status.

### GSEA Analysis of High and Low Expression of ASF1B in Pan-Cancer

**Figure 8:**
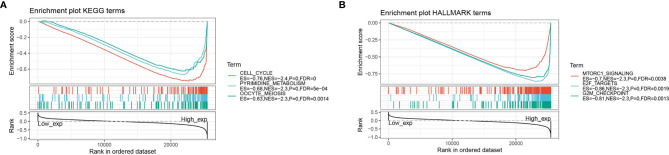
GSEA analysis of ASF1B; **(A)** enrichment analysis of ASF1B in KEGG signaling pathway; **(B)** enrichment analysis of ASF1B in HALLMARK signaling pathway.

The GSEA enrichment analysis revealed that ASF1B was involved in the regulation of many cancer metabolics and cancer immune signaling pathways. The three KEGG signaling pathways most significantly associated with ASF1B high expression were shown in **Figure 8A**, where ASF1B high expression was significantly enriched in cell cycle, pyrimidine metabolism, and oocyte meiosis-related pathways. The three HALLMARK pathways most significantly associated with ASF1B high expression were shown in **Figure 8B**, where ASF1B high expression was positive in MTORC1 signaling system, E2F target, and G2M checkpoint-related pathways.

## Discussion

The identification of cancer prognostic biomarkers can aid the prediction of the prognostic status of each patient, which may help to achieve personalized cancer treatment (12). It has been partially demonstrated that some key genes can influence development of PTC. For example, mutations in BRAF and RAS loci are closely associated with development of PTC; rearrangements involving RET and NTRK1 can activate the MARK signaling pathway, accelerating the progression of PTC. With the development of bioinformatics, extensive studies of microarrays and RNA-seq have made it possible to discover new biomarkers for PTC (13). He et al. (14) reported that the discovery of CDH3, CTGF, CYR61, FGF13, CHRDL1, and OGN was not possible without comprehensive bioinformatics analysis and multiple datasets in immune related pathways that were closely involved in PTC tumorigenesis and prognosis. In a whole, there is a need to find more biomarkers that can more accurately predict cancer and its prognosis. To focus on the role and value of the biomarker in other cancers is to provide ideas for cancer therapeutic targets, in addition to fully understand the mechanism of PTC progression.

In this study, a total of 12 key genes were obtained by a crossover of differentially upregulated genes in THCA and the genes associated with disease-free survival in THCA. ASF1B gene with the highest risk coefficient was selected to observe its expression and predictive value in THCA prognosis as well as in a variety of other cancers. ASF1B, preferentially involved in cell proliferation, is an isoform of the histone chaperone protein ASF1, which mainly affects DNA replication, DNA damage repair, and transcriptional processes *via* chromatin function regulation. This study showed that ASF1B was highly expressed in the majority of cancers. It supported previous studies that ASF1B expression was elevated in cervical and breast cancers (15, 16). Moreover, elevated ASF1B expression was associated with not only poor prognosis in human lung adenocarcinoma but also the diagnosis and prognosis of breast, renal cell, and cervical cancers (16, 17); In this study, Kaplan-Meier curve demonstrated that high ASF1B expression was associated with poor cancer prognosis in 13 types of cancer, which suggested that ASF1B may serve as a prognostic predictive marker for THCA and a potential predictor of other cancers.

In addition, this study found that ASF1B expressions in THCA, KIRC, LGG, and LIHC were significantly positively correlated with the infiltration levels of B cells, CD4+ T cells, CD8+ T cells, Neutrophils, Macrophage, and Dendritic cells. It’s suggested that ASF1B may alter the tumor microenvironment or the degree of tumor immune cell infiltration. In general, the process of immunity is mainly regulated through T cells, which can recognize tumor antigens in the tumor microenvironment and deliver tumor antigens to T cell receptors *via* antigen-presenting cells. Impaired T-cell function in most cancer patients is the main reason why cancer cells can evade protective anti-tumor immunity. Based on our findings, the mechanism of ASF1B regulation of immunity, although not yet confirmed experimentally, is theoretically feasible with ASF1B inhibitors. Some studies have reported that ASF1B may induce differentiation of sarcomatoid phenotype as a prognostic indicator by regulating the microenvironment and epithelial-mesenchymal transition in malignant mesothelioma (18). In further analysis it’s also found that ASF1B may regulate tumor immune patterns in various tumors by regulating the expression of specific immune checkpoint genes. Thanks to immune checkpoints, immunization is increasingly being used in the field of tumor immunotherapy with some significant breakthroughs. In many cancers such as non-small cell lung cancer (19), melanoma (20), renal cell carcinoma (21), and hepatocellular carcinoma (22), immune checkpoint inhibitors such as CTLA4 inhibitors and PD-1 inhibitors have been shown to have significantly positive effects on patient survival.

Previously study found that ASF1B may change the tumor microenvironment and changes the percentage of the macrophage and some other immune cells such as dendritic cell, monocyte and T cells regulatory (Tregs) (19). ASF1B was found to be associated with immune cell infiltration in HCC (23). ASF1B plays a key role in modifying the chromatin nucleosome structure by maintaining continuous supply of the histones at nucleosome assembly sites as well. Packaging of nucleosomal DNA into distinct structures relies on complex interplays between modifications of histones and DNA, histone variants, chromatin-binding proteins, and noncoding RNAs (24).

The DNA mismatch repair system is a type of safety and security systems that can repair DNA base mismatches in human cells. It has an important role in maintaining the integrity and stability of genetic material and avoiding the generation of genetic mutations (25). In this study, five DNA repair genes MLH1, MSH2, MSH6, PMS2, and EPCAM were evaluated in detail. It’s found that ASF1B expression was significantly positively correlated with DNA repair genes in most cancers. In addition, ASF1B expression was significantly and positively correlated with methyltransferases in many cancer species. However, up till now rare studies have been reported on mechanisms between ASF1B expression and DNA methylation, and this study sheds lights on ASF1B expression and DNA methylation for future investigations.

Finally, GSEA enrichment was used to analyze the biological functions of ASF1B in tumors and it was found that the highly expressed ASF1B was mainly enriched in cell cycle, oocyte meiosis, pyrimidine metabolism, and other related pathways, and was positive in MTORC1 signaling system, E2F targets, and G2M checkpoint related pathways. E2F transcription factor family is one of the most important cytokines involved in cell cycle G1-S phase. E2F transcription factors not only regulate the expression level of target genes but also ensure target genes are largely transcribed in a cell cycle-dependent manner. Therefore, abnormalities in E2F transcription factors play a role in tumorigenesis. Also, E2F transcription factors have been found to regulate ASF1B (26). The ASF1B protein is a substrate for the regulation of cell cycle kinase class (27, 28). The cell cycle kinase plays a key role in regulating chromatin nucleosome structure by ensuring a constant supply of histones at the nucleosome assembly site while interacting directly with transcriptional regulators (29, 30). In addition, it has also been reported that H3K56ac is a key regulator of chromatin assembly/disassembly responses mediated by the histone chaperone Asf1, which is regulated by mTORC1 signaling (31, 32). Our pan-cancer analysis similarly supported the function of ASF1B in the regulatory mechanism.

## Conclusion

In summary, we analyzed the transcriptomic data of cancers in public databases, and found some new information from differentially expressed genes, especially the upregulated expression of ASF1B gene correlated to the prognosis of thyroid cancer (THCA), and ASF1B is associated with disease prognosis in THCA and can be used as a biomarker for THCA prognosis. In the pan-cancer analysis, ASF1B expression is related with immune cell infiltration level and the tumor microenvironment and affects cancer development by interrupting cell cycle, interfering with DNA mismatch repair, promoting DNA methylation, etc. Future study should work on providing a bioinformatics basis for the mechanism of ASF1B in tumor immunity and tumor microenvironment. However, some limitations should be acknowledged in this study. The above conclusions are obtained *via* bioinformatics analysis merely, further biological experiments are required to confirm the function and molecular mechanisms of ASF1B and its signal pathways in THCA.

## Data Availability Statement

The original contributions presented in the study are included in the article/**Supplementary Material**. Further inquiries can be directed to the corresponding author.

## Author Contributions

The authors have contributed equally to this work and share first authorship. All authors contributed to the article and approved the submitted version.

## Conflict of Interest

The authors declare that the research was conducted in the absence of any commercial or financial relationships that could be construed as a potential conflict of interest.

## Publisher’s Note

All claims expressed in this article are solely those of the authors and do not necessarily represent those of their affiliated organizations, or those of the publisher, the editors and the reviewers. Any product that may be evaluated in this article, or claim that may be made by its manufacturer, is not guaranteed or endorsed by the publisher.

## Supplementary Material

Supplementary Figure 1 Differentially expressed genes and functional enrichment analysis; **(A)** volcano plot: black dots indicate significantly differentially up-regulated genes and yellow dots indicate significantly differentially down-regulated genes; **(B)** heat map: differential gene expression heat map, where different colors represent expression trends in different tissues.

Supplementary Table 1 List of datasets.

Supplementary Table 2 The gene lists of DEGs and Genes about DFS.

## Abbreviations

ACC, Adrenocortical Carcinom; BLCA, Bladder Urothelial Carcinoma; BRCA, Breast invasive carcinoma; CESC, Cervical squamous cell carcinoma and endocervical adenocarcinoma; CHOL, Cholangiocarcinoma; COAD, Colon adenocarcinoma; DLBC, Lymphoid Neoplasm Diffuse Large B-cell Lymphoma; ESCA, Esophageal carcinoma; GBM, Glioblastoma multiforme; HNSC, Head and Neck squamous cell carcinoma; KICH, Kidney Chromophobe; KIRC, Kidney renal clear cell carcinoma; KIRP, Kidney renal papillary cell carcinoma; LAML, Acute Myeloid Leukemia; LGG, Brain Lower Grade Glioma; LIHC, Liver hepatocellular carcinoma; LUAD, Lung adenocarcinoma; LUSC, Lung squamous cell carcinoma; MESO, Mesothelioma; OV, Ovarian serous cystadenocarcinoma; PAAD, Pancreatic adenocarcinoma; PCPG, Pheochromocytoma and Paraganglioma; PRAD, Prostate adenocarcinoma; READ, Rectum adenocarcinoma; SARC, Sarcoma; SKCM, Skin Cutaneous Melanoma; STAD, Stomach adenocarcinoma; TGCT, Testicular Germ Cell Tumors; THCA, Thyroid carcinoma; THYM, Thymoma; UCEC, Uterine Corpus Endometrial Carcinoma; UCS, Uterine Carcinosarcoma; UVM, Uveal Melanoma; PTC, Papillary thyroid carcinoma; TCGA, The cancer genome atlas; GTEx, The Genotype-Tissue Expression; DEGs, Differentially expressed genes; GO, Gene ontology; KEGG, Kyto encyclopedia of genes and genomes; OS, Overall survival; DFS, Disease free survival; KM, Kaplan Meier.
