# Supplementary figures and images for: Comprehensive Pan-Cancer Analysis and the Regulatory Mechanism of ASF1B, a Gene Associated With Thyroid Cancer Prognosis in the Tumor Micro-Environment
Source: Front Oncol. 2021 Aug 20;11:711756. doi: 10.3389/fonc.2021.711756 (PMC8417739; doi:10.3389/fonc.2021.711756)

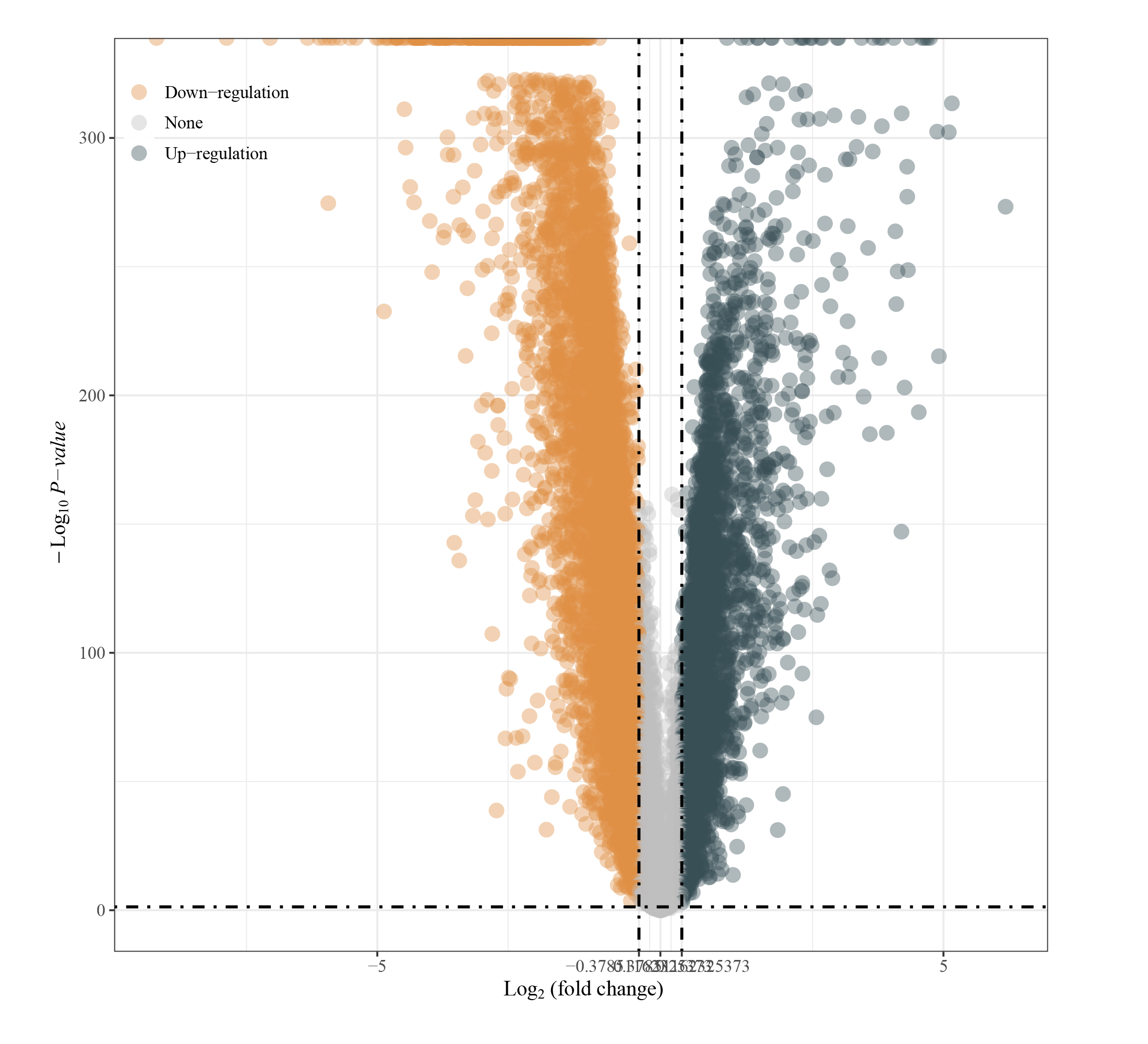

Supplement: Supplementary Figure 1 — Differentially expressed genes and functional enrichment analysis; (A) volcano plot: black dots indicate significantly differentially up-regulated genes and yellow dots indicate significantly differentially down-regulated genes; (B) heat map: differential gene expression heat map, where different colors represent expression trends in different tissues. [file Image_1.jpeg]
